# A phase IIa proof-of-concept, placebo-controlled, randomized, double-blind, crossover, single-dose clinical trial of a new class of bronchodilator for acute asthma

**DOI:** 10.1186/s13063-018-2720-6

**Published:** 2018-06-18

**Authors:** Veronica Swystun, Francis H. Y. Green, John H. Dennis, Emmanouil Rampakakis, Gurkeet Lalli, Morenike Fadayomi, Andrea Chiu, Grishma Shrestha, Sharif Galal El Shahat, David Evan Nelson, Tamer Y. El Mays, Cora A. Pieron, Richard Leigh

**Affiliations:** 1 Department of Medicine, University of Calgary, Calgary, AB Canada; 2 Department of Pathology & Laboratory Medicine, University of Calgary, Calgary, AB Canada; 3 SolAeroMed Inc, 120-4838 Richard Rd SW, Calgary, AB T3E 6L1 Canada; 4 JSS, Montreal, QC Canada

**Keywords:** Novel bronchodilator, Asthma, S1226, Carbon dioxide (CO_2_), Perflubron, Clinical trial

## Abstract

**Background:**

This study evaluates a novel bronchodilator, S1226, for its efficacy in reversing allergen-induced bronchoconstriction in subjects with mild, allergic asthma. S1226 is a new class of bronchodilator that is an aerosol/vapor/gas mixture combining pharmacological and biophysical principles for a novel mode of action. It contains a potent bronchodilator gas (carbon dioxide or CO_2_) and nebulized perflubron (a synthetic surfactant possessing mucolytic properties). It has demonstrated rapid reversal of allergen-induced bronchoconstriction in an ovine study model.

**Methods:**

This was a phase IIa proof-of-concept, placebo-controlled, randomized, double-blind, crossover single-dose clinical trial to evaluate the safety, tolerability, and efficacy of S1226 (8% CO_2_) administered by nebulization following an allergen-induced early asthmatic response in 12 subjects with mild, allergic asthma. Primary safety endpoints were adverse events, vital signs, pulse oximetry, and spirometry. Efficacy endpoints included bronchodilator response (measured as the forced expiratory volume in 1 s or FEV_1_) over time, the area under the curve of FEV_1_ for the early asthmatic response over time, and achievement of responder status, defined as a 12% improvement after the allergen challenge.

**Results:**

No significant safety issues were observed. All adverse events were non-serious, mild, and transient. There was a statistically significant decrease in peripheral blood oxygenation levels over time in the placebo group following allergen inhalation, whereas blood oxygenation was maintained at normal levels in the S1226-treated subjects (*P* = 0.028). This effect was greatest 5 min after start of treatment (*P* < 0.001). The recovery rate was faster but not significantly so (*P* = 0.272) for S1226 compared to the placebo at earlier time points (5, 10, and 15 min), as assessed by ≥12% reversal of FEV_1._ The recovery of FEV_1_ over time was significantly greater (*P* = 0.04) with S1226 compared to the placebo.

**Conclusions:**

S1226 was safe, tolerated well, and provided bronchodilation and improved blood oxygenation in subjects with mild atopic asthma following allergen-induced bronchoconstriction. Additional studies to optimize the therapeutic response are indicated.

**Trial registration:**

ClinicalTrials.gov, NCT02334553. Registered on 12 November 2014.

**Electronic supplementary material:**

The online version of this article (10.1186/s13063-018-2720-6) contains supplementary material, which is available to authorized users.

## Background

Inhaled bronchodilators, including short-acting beta-2-adrenergic agonists and anticholinergics along with anti-inflammatory medications, are first-line treatments for emergency management of acute asthma exacerbations [1, 2]. Effective bronchodilation is a challenge during exacerbations and having an additional class of bronchodilator in the treatment arsenal would be beneficial.

Existing bronchodilator treatments are effective in most cases, but there are several reasons why they can fail during exacerbations. First, in severe exacerbations, particulate medications may be unable to penetrate airways obstructed by bronchoconstriction, mucus plugs, and inflammation. Second, patients become less responsive to traditional bronchodilators from chronic use, due to tachyphylaxis and tolerance [3–5]. Third, there is a group of patients with asthma refractory to standard therapies [6] and these patients represent an unmet clinical need for whom alternative treatment options are required. The challenge is to find an alternative treatment to augment conventional therapy.

This study evaluates a new class of bronchodilator, S1226, which combines pharmacological and biophysical properties. S1226 is an aerosol/vapor/gas mixture containing a bronchodilator gas (CO_2_) and a nebulized synthetic surfactant (perflubron) that has mucolytic [7, 8] and anti-inflammatory properties [9–11]. Previous studies have shown that inhalation of 6% CO_2_ over 4–5 min in atopic asthmatics relieved exercise-induced asthma. However, its effects were short-lived [12]. The bronchodilatory effects of CO_2_ are complex. They involve pH and epithelial-dependent mechanisms [13] and do not require adrenergic [14] or cholinergic pathways [12]. The combination of CO_2_ with perflubron in preclinical studies produced a synergistic effect, providing rapid and sustained bronchodilation [13–15]. S1226 showed rapid opening of airways in sheep sensitized and challenged with house dust mites and rats sensitized and challenged with ovalbumin. The effect was fast (<4 s for bronchodilation) and sustained (>20 min) [15].

A phase I clinical trial was completed in 36 healthy volunteers to assess safety and tolerability of S1226. All adverse events (AEs) were mild and transient and S1226 appeared safe at CO_2_ concentrations of 4%, 8%, and 12% [16].

We now undertook a phase IIa, proof-of-concept, placebo-controlled, randomized, double-blind, crossover single-dose study to demonstrate safety, tolerability, and efficacy of nebulized S1226 (8% CO_2_) in subjects with mild, atopic asthma. We chose 8% CO_2_ in this first study in asthmatic humans as this level of CO_2_ was effective in animal studies and had no AEs in the phase I clinical trial [16]. An abstract of the study described in this paper was presented as a poster at the American Thoracic Society meeting in 2016 [17].

## Methods

### Participants

Adult volunteers, aged 18–40 years, with mild, allergic asthma were screened to confirm the presence of an early asthmatic response (EAR) to inhaled allergen. PC_20_ methacholine indicates a provocative concentration of inhaled methacholine producing a 20% reduction in forced expiratory volume in 1 s (FEV_1_). All subjects had had asthma for more than 3 months, and their inclusion was based on a PC_20_ methacholine of ≤16 mg/mL and an allergen-induced EAR producing a ≥20% reduction in FEV_1_ during screening. The full list of inclusion and exclusion criteria are listed in Additional file 1.

### Trial design

**Fig. 1:**
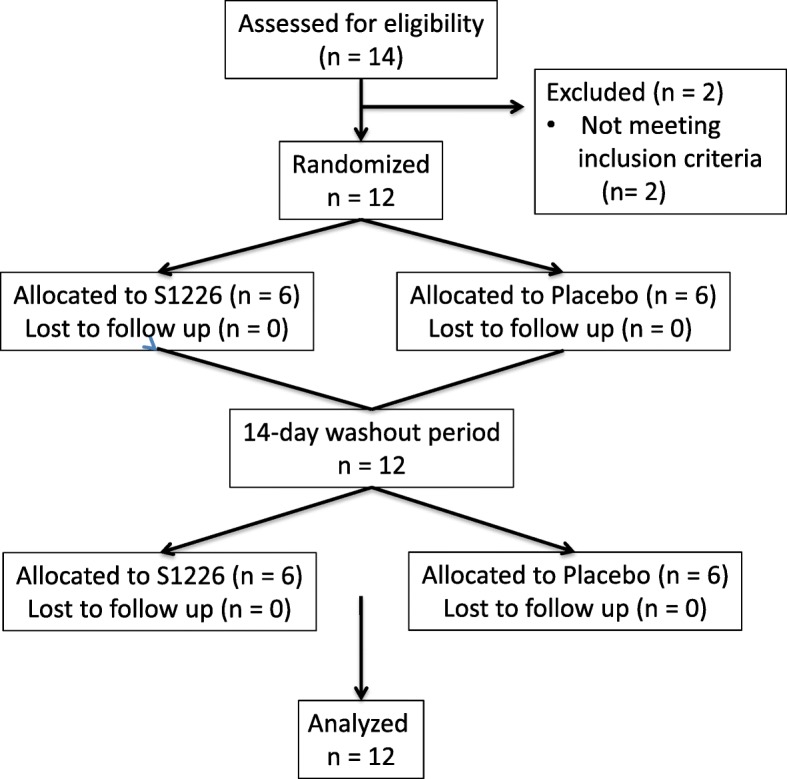
Study design from enrollment to analysis following the CONSORT guidelines

This was a phase IIa, randomized, double-blind, placebo-controlled, crossover study enrolling 12 subjects with asthma. The study design is outlined in Fig. 1.

The study consisted of a <30-day screening period, two treatment periods with a minimum 2-week washout in between, and a follow-up visit 1 day later. A detailed schedule of assessments during the clinical trial study are shown in Additional file 2. Baseline screening involved routine chemistry, hematology, urinalysis, methacholine inhalation testing (PC_20_ ≤ 16 mg/mL), and allergen skin prick tests. An allergen inhalation challenge to common aero-allergens (cat, horse, and grass pollen) was used to establish the allergen concentration required to achieve a 20% fall in FEV_1_ as previously reported [5, 18]. This concentration was used in the subsequent treatment periods with the goal of achieving comparable EARs (20% fall in FEV_1_) between the two treatment periods. The allergen challenge model has excellent within-subject repeatability and hence, in crossover studies, a sample size of 12 subjects is sufficient to detect an effect on clinically relevant outcome measures with a power of 90% [18].

For this study, a sample size of 16 subjects was estimated to be able to detect a treatment difference of at least a 14% change in FEV_1_ with a 95% confidence interval with a power of over 90% in a two-sided test, assuming that the variability observed would be like the response to S1226 in the sheep allergen challenge model [15]. The sheep model used airway resistance as a model of airway caliber. Under similar assumptions, a sample size of 12 subjects was estimated to able to detect a treatment difference of at least 17% change in FEV_1_ with a 95% confidence interval and a power of 90%.

In summary, in this proof-of-concept study, using the animal data and taking the robustness of this model into consideration, our goal was to recruit up to 16 subjects with the expectation of retaining at least 12 subjects to account for any dropouts.

The test procedures were conducted at least 6 weeks after any relevant seasonal allergen exposure and the study lasted approximately 9 weeks for each subject.

Each treatment period consisted of a methacholine challenge on day 1 to confirm airway hyperresponsiveness (methacholine PC_20_ within 1 doubling concentration of baseline PC_20_), an allergen challenge followed by study intervention (S1226 or placebo) on day 2, and spirometry on day 3 to confirm recovery from allergen inhalation. Immediately after a 20% fall in FEV_1_ was documented as part of the EAR, a single dose of S1226 (8%) or placebo was administered via nebulization to subjects over 2 min with a Circulaire ® II hybrid nebulizer system (Westmed, Tucson, AZ, USA). Subjects were allowed to stop receiving the interventions at any time.

S1226 (8%) (SolAeroMed, Calgary, AB, Canada) consists of 3 mL of perflubron nebulized with medical grade compressed gas (Praxair Canada, Mississauga, ON, Canada) containing 8% CO_2_, 21% O_2_, and balanced N_2_ at a flow rate of 9 L/min. The placebo was 3 mL saline (0.9% NaCl) (Smiths Medical ASD Inc. Markham, ON, Canada) with medical grade air (Praxair Canada, Mississauga, ON, Canada) at a flow rate of 9 L/min. The Circulaire nebulizer system incorporates an inflatable reservoir bag, which collects nebulized gas during exhalation so that during inhalation the subject receives both freshly generated nebulized drug and stored nebulized drug.

The study was double-blind. The treatments were prepared and administered by an unblinded research associate, who performed no other procedures in the study. Personnel conducting testing procedures and collecting study outcomes were blinded to the treatment allocation. Although S1226 (8%) and placebo have different viscosities and densities [16], they had identical visual appearances and the same model of nebulizer was used to administer both compounds. Thereby, the identity of the treatments remained blinded to the study subjects.

The treatment order of placebo and S1226 (8%) for each subject was randomized in a 1:1 ratio in blocks of four, using a computer-generated randomization list. Both study subjects and the clinical personnel involved in collection and monitoring of study data and evaluation of AEs were blinded with respect to the subject’s treatment allocation.

All procedures were conducted in accordance with the standard operating procedures for the Respiratory Clinical Trials Centre. Calgary Lab Services completed the clinical laboratory work. The project was monitored by and the statistical analysis was completed by JSS Medical Research Inc., St Laurent, QC, Canada.

The study was conducted at the Respiratory Clinical Trials Centre, University of Calgary, AB, Canada, from January 2015 to April 2016, in accordance with the Declaration of Helsinki (2014). The Conjoint Health Research Ethics Board of the University of Calgary approved the study protocol, amendments, and consent form (REB14–1581). Written informed consent was obtained from all subjects.

### Outcome measures

The primary outcome was safety; efficacy was a secondary outcome measure. The incidence of treatment-emergent AEs was monitored, recorded, and graded for severity and assigned attribution.

The safety and tolerability of S1226 (8%) was evaluated based on the following assessments:

- The number and proportion of subjects experiencing treatment-emergent AEs
- 12-lead electrocardiogram at screening and follow-up
- Vital signs (heart rate, respiratory rate, and blood pressure) at screening, pre-dose, and at 120 min post-dose
- Pulse oximetry at screening, pre-dose, and at 5, 10, 15, 20, 30, 45, 60, and 90 min post-dose and follow-up
- Safety biochemistry, hematology, and urinalysis at screening and follow-up
- FEV_1_ was measured pre-dose and at 5, 10, 15, 20, 30, 45, 60, and 90 min post-dose, while spirometry (FEV_1_ and forced vital capacity) was conducted at screening and follow-up

The efficacy of S1226 (8%) was evaluated based on the following endpoints:

- Area under curve (AUC) of recovery from the EAR in the first 30 and 90 min following treatment
- Responder status, defined as achieving ≥12% improvement in FEV_1_ after the allergen challenge
- Time to achieve responder status
- Maximum percentage reversal of allergen-induced decrease in FEV_1_ in the first 30 and 90 min following study treatment administration

### Statistical methods

The total number of AEs was summarized for each treatment group by seriousness, intensity, relationship to product, and outcome. AEs were coded using the *Medical Dictionary for Regulatory Activities* (MedDRA version 18.1) and summarized using the total number of AEs, total number and percentage of subjects who experienced an AE, and number and percentage of subjects who experienced an AE within each preferred term. No inferential statistical analysis of safety data was performed.

The AUC of the EAR reversal following study drug inhalation was calculated using the trapezoidal rule. The AUCs within 30 min and 90 min from study drug inhalation were compared between S1226 (8%) and the placebo using a mixed general linear model. The model included subject as a random effect; treatment group, period, and sequence as fixed effects; and FEV_1_ at study drug inhalation as a covariate. A repeated measures analysis of variance (ANOVA) mixed model was used to compare the FEV_1_ and AUC over time between S1226 (8%) and placebo. Like the primary analysis, the model included subject as a random effect; treatment group, period, and sequence as fixed effects; and FEV_1_ at study drug inhalation as a covariate.

Achievement of responder status, defined as ≥12% reversal of FEV_1_ from study drug inhalation within 30 and 90 min, was compared between S1226 (8%) and the placebo using logistic regression where period and FEV_1_ at study drug inhalation were entered as terms. Time to achieve responder status was compared between treatment groups using a Cox regression. The model considered treatment group, period, and FEV_1_ at study drug inhalation as covariates. In addition, randomization order was included as a covariate in the mixed models of AUC_30_, AUC_90_, FEV_1_, and AUC over time to evaluate a carryover effect that would favor one treatment vs the other.

## Results

A total of 14 subjects were screened, of whom 12 were eligible and received both interventions. One subject missed the final follow-up visit but their assessments for the two intervention treatments were included in the results.

Patient characteristics at baseline, including demographic and clinical measurements, are summarized in Table 1. The mean (standard deviation, SD) age at baseline for the total cohort was 27.3 (2.4) years. Exactly half of the subjects were female and Caucasian; Asians accounted for 25.0% of subjects. Mean (SD) height, weight, and body mass index (BMI) were 1.7 (0.1) m, 68.5 (17.9) kg, and 23.8 (4.5) kg/m^2^, respectively.

**Table 1:** Baseline patient demographics and clinical characteristics

Variable	Measure	Total (*N* = 12)
Age (years)	Mean (SD)	27.3 (2.38)
	Median	27.0
	Range	23.0–31.0
	95% confidence interval	(25.7, 28.8)
Gender	Female	6 (50.0%)
	Male	6 (50.0%)
Race	Asian	3 (25.0%)
	Caucasian	6 (50.0%)
	Other	3 (25.0%)
Height (m)	Mean (SD)	1.7 (0.11)
	Median	1.7
	Range	1.6–1.9
	95% confidence interval	(1.6, 1.8)
Weight (kg)	Mean (SD)	68.5 (17.91)
	Median	61.4
	Range	46.1–113.3
	95% confidence interval	(57.1, 79.9)
BMI (kg/m^2^)	Mean (SD)	23.8 (4.47)
	Median	23.2
	Range	18.6–35.8
	95% confidence interval	(20.9, 26.6)

*BMI* body mass index, *SD* standard deviation

### Safety and tolerability

During the study, 17 AEs were reported, all of which were judged to be mild in severity and transient. Of the 17 AEs, 10 were not related to the investigational products. The majority of these (*n* = 8) were related to the nebulizer device. Two AEs were possibly related to the placebo and five AEs were probably related to S1226. AEs associated with S1226 were palpitations (1), chest discomfort (1), dizziness (2), and anxiety (1), while feeling hot (1) and flushing (1) were associated with the placebo. Details of the AEs by severity, relation to drug, outcome, and preferred terms are given in Additional files 3 and 4.

No abnormalities in the electrocardiogram, vital signs (respiratory rate and blood pressure), and biochemistry variables were associated with S1226 or the placebo at screening, pre-dose, or 120 min post-dose.

**Fig. 2:**
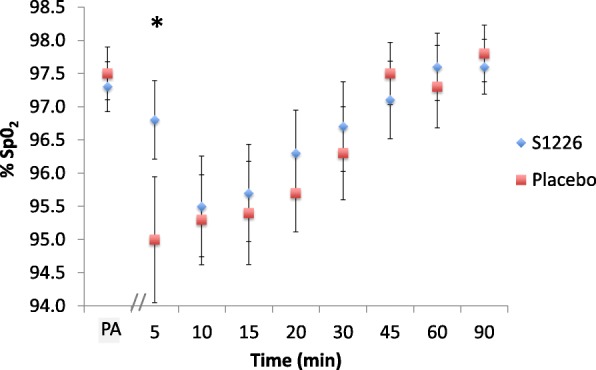
Oxygen saturation measured by pulse oximetry, placebo vs S1226. The absolute change between pre-dose and post-dose %SpO_2_ levels was significantly different (*P* = 0.028) for the S1226 group compared with the placebo group over time. %SpO_2_ significantly decreased 5 min post-treatment following the allergen challenge for the placebo *P < 0.001 but not for S1226 (*P* = 0.267). Error bars show standard errors. PA pre-allergen

When using a mixed model that adjusted for treatment period, sequence, and pre-dose percentage oxygen saturation measured by pulse oximetry (%SpO_2_), the absolute change between pre-dose and post-dose %SpO_2_ was significantly different (*P* = 0.028) for the S1226 group compared with the placebo group over time (Fig. 2). Least squares estimates showed that the S1226 group experienced a lower drop in %SpO_2_ (estimate: -0.627; *P* = 0.267) compared to the placebo group (estimate: -2.502; *P* < 0.001) 5 min post-dose. Furthermore, the placebo group showed higher absolute decreases in %SpO_2_ for all time points with the exceptions of the 45-min time point.

### AUC and FEV_1_ over time

**Fig. 3:**
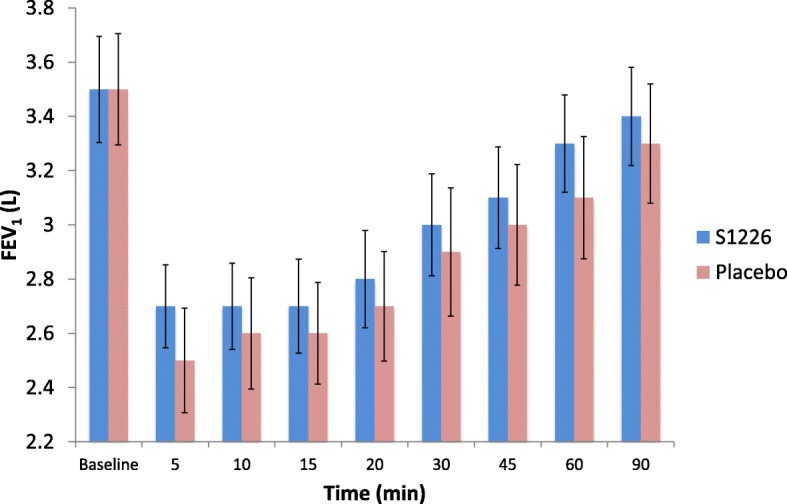
Change in FEV_1_ over time. Baseline adjusted FEV_1_ over time for S1226 was significantly higher than placebo (*P* = 0.043). Error bars show the standard deviation

FEV_1_ over time was significantly greater for S1226 compared with the placebo, with the difference becoming apparent as early as 5 min post-treatment (*p* = 0.043) (Fig. 3). There were no statistically significant differences for AUC, although values for FEV_1_ were numerically higher after S1226 for all time points. The mean (95% confidence interval, CI) adjusted difference in FEV_1_ AUC (0–30 min) between S1226 and the placebo was 0.406 (−6.146, 6.958; *P* = 0.893) while the mean (95% Cl) in FEV_1_ AUC (0–90 min) was 1.509 (−18.973, 21.991; *P* = 0.873). There were no significant carryover effects that would favor one treatment vs the other.

### Achievement of responder status

**Fig. 4:**
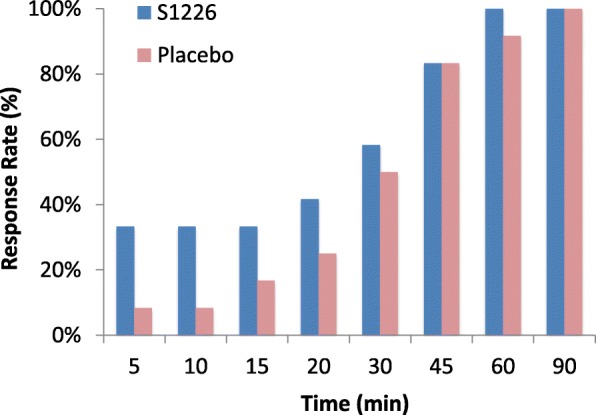
Time to achieve 12% reversal (response) in FEV_1_ following S1226 or the placebo (*P* = 0.272)

The recovery rate was faster for S1226 than the placebo at earlier time points (5, 10, and 15 min) when assessing ≥12% reversal of FEV_1_ (Fig. 4). Time to achieving responder status within the 30-min period was not different between the two treatments (*P* = 0.272).

## Discussion

A single dose of S1226 (8%) in subjects with mild, allergic asthma was safe and tolerated well and produced moderate bronchodilation following allergen-induced bronchoconstriction. Both components of S1226 (perflubron and CO_2_ ) have undergone extensive clinical testing and their safety profiles are well documented [9, 19, 20]. Subjects receiving S1226 reported more AEs than those receiving the placebo, but these were mild in severity, they were transient, and the majority were related to the nebulization device and not to S1226. These results are similar to those from the phase I clinical trial [16]. Based on previous research, AEs related to S1226 (dizziness, anxiety, and chest discomfort) are known transient side effects of CO_2_ inhalation [19, 20]. Perflubron is a stable compound used extensively in clinical applications such as bronchial lavage, liquid ventilation, and gastrointestinal contrast imaging with no known toxic effects [9].

With respect to efficacy, although the response to S1226 was numerically better compared to the placebo for all endpoints, only FEV_1_ over time demonstrated a statistically significant improvement in comparison to the placebo. We cautiously chose 8% CO_2_ (rather than 12%) as the concentration of CO_2_ and a treatment duration of 2 min. It is likely, based on the preclinical studies, that efficacy would be improved with the higher dose of CO_2_ or with an increased duration of nebulization.

S1226 was significantly more effective than the placebo at maintaining our subjects’ oxygenation saturation levels. Although the changes in oxygen saturation in our study are not clinically significant, because we necessarily studied subjects with mild and controlled asthma, the results demonstrate the potential for improving oxygenation in severe asthma exacerbations with this experimental therapy.

Six subjects experienced difficulty breathing through the nebulization device because of the volume limitation of the reservoir bag. This was not considered to be related to the drug per se. However, in the S1226 group, this difficulty may be attributed to, in part, the increased ventilatory drive caused by inhaled CO_2_ [20]. In future studies, ease of breathing and efficacy could be improved by using a facemask for delivery. A potential advantage of the latter is that there are CO_2_ receptors in the nasal passages that might augment the CO_2_ bronchodilator response [21].

S1226 has a unique mechanism of action, activating both pharmacological and biophysical pathways, differentiating it from traditional treatments [12–14]. Bronchoscopic studies in sheep have shown that S1226 dilates constricted airways within seconds, suggesting that it activates neural mechanisms, most likely through non-cholinergic, non-adrenergic nerve receptors located on and between epithelial cells in the airways [13–15]. Animal studies show S1226 to be equally effective for treating early and late phase asthmatic responses [14, 15, 17].

Perflubron can absorb twice its volume of CO_2_, thus increasing the effective dose of CO_2_ to the airways. This property may account for its synergistic effect when combined with CO_2_. Furthermore, perflubron has surfactant and mucolytic properties, which allow it to spread rapidly through airways [7, 22].

A portable rescue device is being developed for delivering S1226 that can be used in emergency home-care situations. S1226 also has potential for treating diseases such as chronic obstructive pulmonary disease and cystic fibrosis, for which rapid bronchodilation and mucous clearance are required. Due to its biophysical properties, it has the potential to enhance the delivery of other drugs to diseased or inflamed areas of the lung.

## Conclusions

In conclusion, this study shows that S1226 is safe and effective in subjects with mild asthma and warrants further investigation in a larger patient sample. In view of S1226’s rapid effect, alternative mechanism of action, and potential to penetrate obstructed airways, it could provide additional bronchodilation when current standard-of-care rescue medication is failing.

## Additional files

Additional file 1: Inclusion and exclusion criteria for participants enrolled in the study (DOCX 18 kb)

Additional file 2: Schedule of assessments during the clinical trial study. (DOCX 102 kb)

Additional file 3: Adverse events (AEs) by severity, relation to drug, and outcome. (DOCX 14 kb)

Additional file 4: Incidence of adverse events (AEs). (DOCX 15 kb)

Additional file 5: CONSORT 2010 checklist. (DOC 218 kb)

## Abbreviations

AE: Adverse event
ANOVA: Analysis of variance
AUC: Area under curve
BMI: Body mass index
CI: Confidence interval
CO_2_: Carbon dioxide
EAR: Early asthmatic response
FEV_1_: Forced expiratory volume in 1 s
PC_20_: Provocative concentration of inhaled methacholine producing a 20% reduction in FEV_1_
SD: Standard deviation
SpO_2_: Oxygen saturation measured by pulse oximetry
