# Supplementary material for: A phase IIa proof-of-concept, placebo-controlled, randomized, double-blind, crossover, single-dose clinical trial of a new class of bronchodilator for acute asthma
Source: Trials. 2018 Jun 18;19:321. doi: 10.1186/s13063-018-2720-6 (PMC6006836; doi:10.1186/s13063-018-2720-6)
Supplement: Supplementary file 1 — Inclusion and exclusion criteria for participants enrolled in the study (DOCX 18 kb) [file 13063_2018_2720_MOESM1_ESM.docx]

**Additional File 1**

**A Phase IIa proof-of-concept, placebo controlled, randomized, double-blind,**

**crossover, single-dose clinical trial of a new class of bronchodilator for acute**

**asthma**

**Inclusion and exclusion criteria for participants enrolled in the study.**

| Inclusion Criteria | Exclusion Criteria |
| --- | --- |
| - Male or female subjects 18-40 years of age. - BMI of 18-40 kg/m^2^. - Subject was not currently on topical or systemic corticosteroids and had not taken any oral/injectable corticosteroid within 60 days prior to study drug administration and had not used any inhaled/nasal corticosteroid within 30 days prior to study drug administration. - Female subjects could not have been pregnant or lactating and must have been practicing an acceptable method of birth control, or be surgically sterile, or postmenopausal. - Subjects must have had asthma for at least 3 months. - Subject was a non-smoker or had not smoked for > 1year and had < 10 pack-year history. - Subject had a methacholine PC_20_ of less than 16 mg/mL. - Subject had normal laboratory values (normal values as clinically judged by the Investigator) for clinical chemistry, hematology, and urinalysis. - Subject was in general good health based on medical history and clinically acceptable results for the following assessments: physical examination, vital signs, and 12-lead ECG, as assessed by study physicians. - Subject was able to communicate effectively with study personnel and was reliable, willing and cooperative in terms of compliance with protocol. | - Any clinically significant abnormality or abnormal laboratory test results found during medical screening or positive test for hepatitis B, hepatitis C, or HIV found during medical screening. - Subjects who required inhaled β_2_-agonist medication more frequently than 4 times a week (other than prophylactically prior to exercise) during the 4 week period before screening. - Subjects who were currently treated with any asthma medication other than inhaled β_2_-agonist. - Subjects with frequent ER visits for asthma, with prior intensive care unit admission or those with prior intubation. - Presence or history of neurologic, endocrine, hepatic, gastrointestinal or kidney disease or therapy that would have jeopardize the subject’s well-being by participating in the study. - Cardiovascular disease that, in the opinion of the Investigator, was not stable or could have put the subject at increased risk by participating in the study. - Any reason which, in the opinion of the Investigator (or delegate), would have prevented the subject from participating in the study. - Clinically significant ECG abnormalities or vital sign abnormalities (systolic blood pressure lower than 90 or over 140 mmHg, diastolic blood pressure lower than 50 or over 90 mmHg, or heart rate less than 50 or over 100 bpm) at screening. - Subject had a history of physician diagnosed panic disorder or other anxiety disorders. - Subject was currently receiving treatment, or had received treatment in the previous 14 days, with monoamine oxidase inhibitors. - Subjects dosed with an investigational drug within 30 days prior to the Screening Visit. - Subjects dosed with biologic therapy within the previous 4 months or 5 half-lives from baseline methacholine testing. - Subject had current (or within the last six months) evidence of alcohol abuse (regularly drinks more than 4 units of alcohol per day; 1 unit = ½ pint of beer, 1 glass of wine, or 1 ounce of spirit). - Positive urine drug screen or urine cotinine test at screening. - Breast-feeding subject. - Positive pregnancy test at screening. - Subject, who in the opinion of the Investigator, was mentally or emotionally unsuitable to participate, or unable/unwilling to comply with the study assessments. |
