# Supplementary material for: A phase IIa proof-of-concept, placebo-controlled, randomized, double-blind, crossover, single-dose clinical trial of a new class of bronchodilator for acute asthma
Source: Trials. 2018 Jun 18;19:321. doi: 10.1186/s13063-018-2720-6 (PMC6006836; doi:10.1186/s13063-018-2720-6)

**Additional File 2**

**A Phase IIa proof-of-concept, placebo controlled, randomized, double-blind, crossover, single-dose clinical trial of a new class of bronchodilator for acute asthma**

**Schedule of assessments during the clinical trial study.**


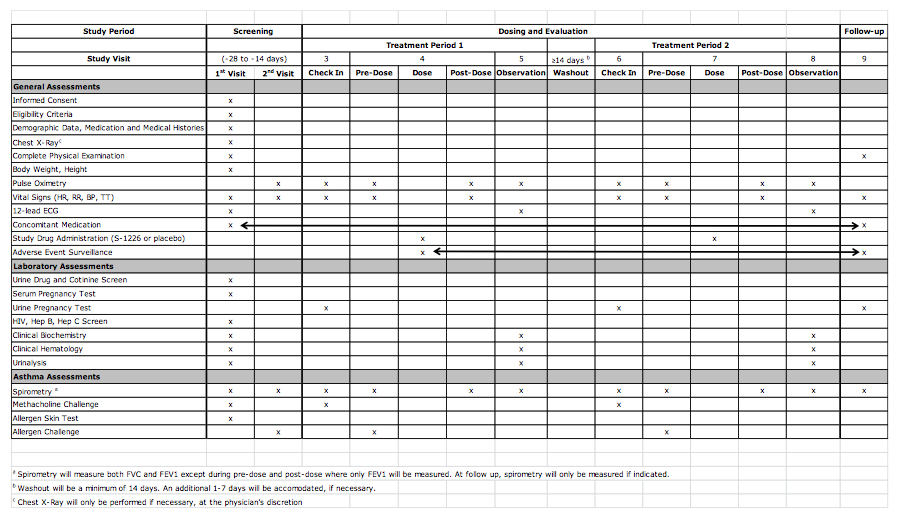

Supplement: Supplementary file 2 — Schedule of assessments during the clinical trial study. (DOCX 102 kb) [file 13063_2018_2720_MOESM2_ESM.docx]
