# Supplementary material for: A phase IIa proof-of-concept, placebo-controlled, randomized, double-blind, crossover, single-dose clinical trial of a new class of bronchodilator for acute asthma
Source: Trials. 2018 Jun 18;19:321. doi: 10.1186/s13063-018-2720-6 (PMC6006836; doi:10.1186/s13063-018-2720-6)
Supplement: Supplementary file 3 — Adverse events (AEs) by severity, relation to drug, and outcome. (DOCX 14 kb) [file 13063_2018_2720_MOESM3_ESM.docx]

**Additional File 3**

**A Phase IIa proof-of-concept, placebo controlled, randomized, double-blind, crossover, single-dose clinical trial of a new class of bronchodilator for acute asthma**

**Adverse Events (AEs) by Severity, Relation to Drug, and Outcome.**

Of the 17 AEs, the majority (n=10) was not related to the investigational products (S1226/placebo), 2 were possibly related, and 5 were probably related. A total of 11 and 4 AEs were reported for S1226 and placebo groups, respectively. A total of 7 (58.3%) subjects in the S1226 group experienced at least one AE compared with 3 (25.0%) subjects in the placebo group. Three (25.0%) subjects in the S1226 group experienced AEs that were judged as probably related to the study drug compared with no subjects in the placebo group, however 1 (8.3%) patient in the placebo group experienced AEs that were possibly related to the drug.

|  | | S-1226 (N=12) | | | Placebo (N=12) | | |
| --- | --- | --- | --- | --- | --- | --- | --- |
|  |  | N of Events | N of Subjects | % of Subjects | N of Events | N of Subjects | % of Subjects |
| TOTAL |  | 11* | 7 | 58.3% | 4* | 3 | 25.0% |
| Severity | Mild | 11 | 7 | 58.3% | 4 | 3 | 25.0% |
| Relationship to drug | Not related | 6 | 6 | 50.0% | 2 | 2 | 16.7% |
|  | Possibly related | 0 | 0 | 0.0% | 2 | 1 | 8.3% |
|  | Probably related | 5 | 3 | 25.0% | 0 | 0 | 0.0% |
| Outcome | Resolved without Sequelae | 11 | 7 | 58.3% | 4 | 3 | 25.0% |

* Two AEs were excluded from this table. One AE occurred during screening (Nervous system disorders: Headache) and one AE occurred during washout (Respiratory, thoracic and mediastinal disorders: Respiratory tract congestion).
