# Supplementary material for: A phase IIa proof-of-concept, placebo-controlled, randomized, double-blind, crossover, single-dose clinical trial of a new class of bronchodilator for acute asthma
Source: Trials. 2018 Jun 18;19:321. doi: 10.1186/s13063-018-2720-6 (PMC6006836; doi:10.1186/s13063-018-2720-6)
Supplement: Supplementary file 4 — Incidence of adverse events (AEs). (DOCX 15 kb) [file 13063_2018_2720_MOESM4_ESM.docx]

**Additional File 4**

**A Phase IIa proof-of-concept, placebo controlled, randomized, double-blind, crossover, single-dose clinical trial of a new class of bronchodilator for acute asthma**

**Incidence of Adverse Events (AEs).**

The AE with respect to system organ class that occurred the most, for a total of 10 events and occurring in 9 (75.0%) subjects, was general disorders and administration site conditions. Within this system organ class, the most frequently reported AE was respiratory complication associated with device (n = 7 for S1226; n = 3 for placebo). Cardiac disorders, specifically palpitations, occurred in 1 (8.3%) patient from the S1226 group compared with 0 subjects from the placebo group. Nervous system disorders and psychiatric disorders, including dizziness and anxiety, respectively, each occurred in 1 (8.3%) patient from the S1226 group compared with 0 from the placebo group. Finally, vascular disorders, specifically flushing, were reported in 1 (8.3%) patient from the placebo group compared with 0 from the S1226 group.

|  | | S-1226 (N=12) | | | Placebo (N=12) | | |
| --- | --- | --- | --- | --- | --- | --- | --- |
| System Organ Class | Preferred Term | N of Events | N of Subjects | % of Subjects | N of Events | N of Subjects | % of Subjects |
| TOTAL AEs |  | 11* | 7 | 58.3% | 4* | 3 | 25.0% |
| Cardiac disorders | Palpitations | 1 | 1 | 8.3% | 0 | 0 | 0.0% |
|  | SUBTOTAL | 1 | 1 | 8.3% | 0 | 0 | 0.0% |
| General disorders and administration site conditions | Chest discomfort | 1 | 1 | 8.3% | 0 | 0 | 0.0% |
|  | Feeling hot | 0 | 0 | 0.0% | 1 | 1 | 8.3% |
|  | Respiratory complication associated with device | 6 | 6 | 50.0% | 2 | 2 | 16.7% |
|  | SUBTOTAL | 7 | 7 | 58.3% | 3 | 3 | 25.0% |
| Nervous system disorders | Dizziness | 2 | 1 | 8.3% | 0 | 0 | 0.0% |
|  | SUBTOTAL | 2 | 1 | 8.3% | 0 | 0 | 0.0% |
| Psychiatric disorders | Anxiety | 1 | 1 | 8.3% | 0 | 0 | 0.0% |
|  | SUBTOTAL | 1 | 1 | 8.3% | 0 | 0 | 0.0% |
| Vascular disorders | Flushing | 0 | 0 | 0.0% | 1 | 1 | 8.3% |
|  | SUBTOTAL | 0 | 0 | 0.0% | 1 | 1 | 8.3% |

* Two AEs were excluded from this table. One AE occurred during screening (Nervous system disorders: Headache) and one AE occurred during washout (Respiratory, thoracic and mediastinal disorders: Respiratory tract congestion).
